# Puerarin protects against acetaminophen‐induced oxidative damage in liver through activation of the Keap1/Nrf2 signaling pathway

**DOI:** 10.1002/fsn3.3609

**Published:** 2023-08-11

**Authors:** Wanhai Zhou, Heng He, Qin Wei, Litao Che, Xin Zhao, Wenwen Liu, Yue Yan, Lianqing Hu, Yonghua Du, Zhongqiong Yin, Yongkang Shuai, Li Yang, Ruizhang Feng

**Affiliations:** ^1^ Sichuan Oil Cinnamon Engineering Technology Research Center Yibin University Yibin China; ^2^ Faculty of Agriculture, Forestry and Food Engineering YiBin University Yibin China; ^3^ Natural Medicine Research Center, College of Veterinary Medicine Sichuan Agricultural University Chengdu China

**Keywords:** acetaminophen, acute liver injury, Keap1/Nrf2, oxidative stress, Puerarin

## Abstract

Puerarin (Pue) is a kind of isoflavone compound extracted from Pueraria lobata, which has significant antioxidant activity. Excessive use of acetaminophen (APAP) can cause oxidative stress in the liver and eventually lead to acute liver injury. The purpose of this study was to investigate the protective effect and the mechanism of puerarin on APAP‐induced liver oxidative damage. In in vitro experiments, puerarin significantly increased the cell activity of HepG2 cells, reduced the ROS accumulation, alleviated the oxidative damage and mitochondrial dysfunction. In in vivo studies, our results showed that puerarin enhanced antioxidant activity and alleviated histopathological damage. Further studies showed that puerarin decreased the expression of Keap1, promoted the nuclear migration of Nrf2, and up‐regulated the expression of GCLC, GCLM, HO‐1 and NQO1. This study demonstrated that puerarin can protect APAP‐induced liver injury via alleviating oxidative stress and mitochondrial dysfunction by affecting the nuclear migration of Nrf2 via inhibiting Keap1.

## Graphical abstract

In this work, We analyzed the protective effect of puerarin against acetaminophen‐induced liver injury in vitro and in vivo and discussed its potential mechanism.

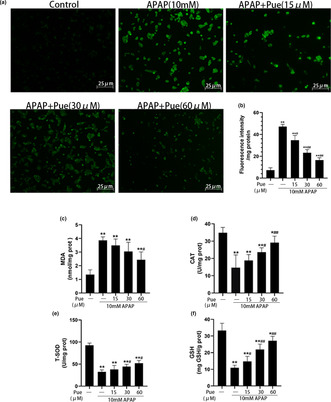

## 1 INTRODUCTION

Acetaminophen (APAP) has significant antipyretic and analgesic effects, making it a common treatment for fever and pain; however, excessive use can lead to serious liver damage (Chun et al., 2009; He et al., 2022). Unfortunately, overuse of APAP has become the leading cause of drug‐induced liver injury (DILI) in many countries around the world (Lee, 2013). Previous studies have shown that the main pathogenic mechanism of APAP‐induced liver injury includes that excessive APAP is converted into a large amount of NAPQI by CYP2E1, which rapidly depletes the intracellular GSH, causing mitochondrial oxidative stress and dysfunction, and finally leading to cell necrosis concentrated in hepatic lobules (Ramachandran & Jaeschke, 2019; Yan et al., 2018). This model of liver injury is widely used in the clinical study of the mechanism of drug‐induced liver injury and potential therapeutic drugs.

Pueraria lobata (*Pueraria montana var. lobata* (*Willd*.) *Sanjappa & Pradeep*) was first recorded in the *Shennong Materia Medical Classic* (Zhao et al., 2018). It has a long history of dual use of medicine and food and has a good reputation as Asian ginseng. According to the *Compendium of Materia Medica* written by Li Shizhen in the Ming Dynasty of China, pueraria lobata has the effect of clearing heat and detoxification and can be used to treat diabetes and reduce liver poisoning caused by alcohol (Liu et al., 2019). It has been shown that the antioxidant effect of pueraria lobata is one of the ways to protect the liver, and its antioxidant activity is positively correlated with the content of isoflavone (Cherdshewasart & Sutjit, 2008; Lee, 2004; Zhao et al., 2010). Puerarin is one of the active components in pueraria lobata and belongs to isoflavone, and as a phytoestrogen, it can be used as a food additive and an alternative medicine (Bacanli et al., 2018; Zhou et al., 2014). Many studies have shown that puerarin has a wide range of pharmacological effects, such as antioxidant (Li et al., 2020), lowering blood lipid (Xu et al., 2021) and anti‐cardiovascular diseases (Li et al., 2017). It has not been reported whether puerarin has protective effect on APAP‐induced acute liver injury. Since oxidative stress plays an important role in the hepatotoxicity of APAP, based on the pharmacological effects of puerarin, we speculate that puerarin may also play a hepatoprotective role in this disease model.

Nrf2 signaling pathway is a classical cellular antioxidant pathway, and its activity is controlled by cytoplasmic protein Keap1 (Kelch‐like ECH associated protein 1) (Nguyen et al., 2009; Xu et al., 2023). It has been reported that Nrf2 knockout mice are highly sensitive to APAP dose compared with wild‐type mice, suggesting that Nrf2 may have a certain resistance to the hepatotoxicity of APAP (Chan et al., 2001; Enomoto et al., 2001). It is reasonable to assume that targeting the Nrf2 signaling pathway can mitigate APAP‐induced liver injury. Therefore, the purpose of this study was to investigate the effect of puerarin on APAP‐induced liver injury, and to explore whether the liver protective mechanism of puerarin is related to the activation of Nrf2 signaling pathway.

## 2 MATERIALS AND METHODS

### 2.1 Chemicals and reagents

Puerarin (98% purity; Product code: S30646) and acetaminophen (99% purity; Product code: S31044) were purchased from Shyuanye (Shanghai, China). Superoxide dismutase (SOD), catalase (CAT), malondialdehyde (MDA), glutathione (GSH), reactive oxygen (ROS), aspartate transaminase (AST), alanine transaminase (ALT), alkaline phosphatase (AKP), γ‐glutamyltransferase (γ‐GT), total bilirubin (TBIL) assay kits were purchased from Nanjing Jiancheng (Nanjing, China). Mitochondrial membrane potential assay kit was purchased from Beyotime (Beijing, China). Total protein extraction reagent and Nuclear and Cytoplasmic Protein Extraction Kit were purchased from Boster (Wuhan, China). The antibodies against Keap1 (10503‐2‐AP), Nrf2 (16396‐1‐AP), GCLC (12601‐1‐AP), GCLM (14241‐1‐AP), HO‐1 (10701‐1‐AP), NQO1 (67240‐1‐Ig) and β‐actin (66009‐1‐Ig) were obtained from Proteintech (Wuhan, China). The antibody against Lamin B (#13435) was obtained from Cell Signaling Technology (Danvers, MA, USA). The secondary antibody was obtained from Boster (Wuhan, China).

### 2.2 Cell culture

The HepG2 cells were purchased from Shanghai Fuheng Biological Cell Bank and cultured in DMEM containing 10% fetal bovine serum with 100 μg mL^−1^ streptomycin and 100 U penicillin, and placed in an incubator with 5% CO_2_ at 37°C.

### 2.3 Cell viability

HepG2 in logarithmic growth phase was taken and culture medium was added to make the cell density of 5 × 10^5^ cells/mL. 100 μL per well was seeded into 96‐well culture plate and cultured overnight in 5% CO_2_ incubator. After 1 h treatment with different concentrations of Pue (0, 3.75, 7.5, 15, 30, 60, 120 μmol L^−1^) in advance, 10 mmol L^−1^ APAP was added. After 24 h of incubation, DMEM medium containing 10%CCK‐8 was added to each well. Then after 1 h of reaction at 37°C, the absorbance (OD) was measured at 450 nm with a microplate analyzer.

### 2.4 Measurement of intracellular ROS

DCFH‐DA is one of the most commonly used and sensitive probes for detecting intracellular reactive oxygen species. After entering cells, it is hydrolyzed into DCFH by esterase. In the presence of reactive oxygen species, it is oxidized into a strong green fluorescent substance that cannot penetrate the cell membrane. HepG2 cells were seeded at 4 × 10^4^ cells per well in a 6‐well plate and placed in an incubator at 37°C 5% CO_2_ overnight. Then, different concentrations of Pue (0, 15, 30, 60 μmol L^−1^) and 10 mM APAP were added and incubated for 24 h. The cell culture medium was added with DCFH‐DA (10 μmol L^−1^) and incubated in darkness for 30 min. Fluorescence microscope was used to take pictures, and then, the fluorescence intensity was measured by a fluorescent enzyme plate. The excitation wavelength was 488 nm, and the emission wavelength was 525 nm.

### 2.5 Measurement of mitochondrial membrane potential

JC‐1 is a fluorescent probe widely used in the detection of mitochondrial membrane potential (ΔΨm). Whenever the ΔΨm is high, JC‐1 forms polymers in the mitochondrial matrix and produces red fluorescence. While at higher ΔΨm, JC‐1 could not gather in the mitochondrial matrix and produces green fluorescence. Therefore, the relative ratio of red‐green fluorescence is often used to measure mitochondrial depolarization. Different treatment groups of HepG2 were incubated for 24 h and 50% JC‐1 working solution was added to each well and cultured at 37°C for 20 min. Fluorescence microscope was used for observation. Then, the cells were collected to prepare cell suspension, and the fluorescence intensity was detected by a luciferase plate analyzer. The detection conditions of JC‐1 polymer were 525 nm excitation light and 590 nm emission light. The detection conditions of JC‐1 monomer were 490 nm excitation light and 530 nm emission light. The degree of ΔΨm depolarization was indicated by the relative proportion of red‐green fluorescence.

### 2.6 Quantitative real‐time PCR

Total RNA was extracted from HepG2 cells and mouse liver using Trizol reagent and reverse transcribed into cDNA. The primers were designed using Oligo7 software according to the target gene sequence in NCBI and were synthesized and purified by Beijing Liuhe BGI Technology Co., Ltd. The primers used in this research are listed in Table 1. According to the recommended annealing temperature of each pair of primers, the temperature gradient was designed to find out the optimal annealing temperature of each gene, and each sample was subjected to RT‐qPCR at the optimal annealing temperature of the above primers. Data were analyzed using the comparative threshold cycle (ΔΔ*C*t) method.

**TABLE 1:** Primer sequences for qPCR.

Gene	Primer sequences fragment size (5′ > 3′) (forward/reversed)
*Keap1*	F‐5′GTGGCTGTCCTCAATCGTCT‐3′
	R‐5′‐TTGCTGTGATCATTCGCCACT‐3′
*Nrf2*	F‐5′TGATATTCCCGGTCACATCGAG‐3′
	R‐5′TGTCCTGTTGCATACCGTCT‐3′
*GCLC*	F‐5′ATCAGTTGGCTACTATCTGTCC‐3′
	R‐5′TACAGATGCAGAAATCACTCCCC‐3′
*GCLM*	F‐5′CATCATCAACTAGAAGTGCAG‐3′
	R‐5′TAATTCCTCCCAGTAAGGCTGT‐3′
*HO‐1*	F‐5′CCTTCCCCAACATTGCCAGT‐3′
	R‐5′CTTGGCCTCTTCTATCACCCTC‐3′
*NQO‐1*	F‐5′CTGAACAAAAGAAGCTGGAA‐3′
	R‐5′TATGAACACTCGCTCAAACCA‐3′
*β‐Actin*	F‐5′ATCAAGATCATTGCTCCTCCTG‐3′
	R‐5′ATACTCCTGCTTGCTGATCCAC‐3′

### 2.7 Animals

SPF ICR mice (6‐week‐old, 18‐22 g) were placed in an environmental control chamber (temperature 23°C, humidity 50%–10%) with a light–dark cycle for 12 h and fed pathogen‐free food and water. The experiment began after 7 days of adaptive feeding. All procedures involving animals and their care in this study were approved (No. 20190604) by the Ethics Committee of Sichuan Agricultural University according to the Regulation of Experimental Animal Management (State Scientific and Technological Commission of the People's Republic of China, No. 2, 1988) and The Interim Measures of Sichuan Province Experimental Animal Management (Science and Technology Bureau of Sichuan, China, No. 25, 2013).

Male mice were randomly divided into five groups, 10 mice in each group, which were blank control group (Ctrl), acetaminophen group (AG), puerarin low‐dose group (Pue_L_), puerarin medium‐dose group (Pue_M_), puerarin high‐dose group (Pue_H_). Mice in Pue group were given 200, 400 and 800 mg kg^−1^ puerarin (dispersed in 0.5% sodium carboxymethyl cellulose) for 10 days, once a day, respectively. Mice in Ctrl and AG were intragastrically given the same amount of 0.5% sodium carboxymethyl cellulose. For 2 h after the last intragastric administration, except for the BC mice, the other mice were intraperitoneally injected with 300 mg kg^−1^ APAP (dissolved in normal saline) and fasted for 24 h.

### 2.8 Liver index assay

The liver index was calculated based on the following formula: mouse liver index = liver weight (mg)/body weight (g).

### 2.9 Hematoxylin–eosin staining

The liver tissues were prepared into paraffin sections, stained with hematoxylin–eosin, and observed histologically under light microscope.

### 2.10 Measurement of blood biochemistry

The blood samples were collected into the blood collection tubes without anticoagulant, placed in an ice box for an appropriate time, centrifuged at 4930 *g* for 10 min, and the serum was collected. The serum was taken to detect the content of AST, ALT, AKP, γ‐GT, and TBIL, and the specific operation method was carried out according to the instructions of the corresponding kit.

### 2.11 Measurement of oxidative stress

Fresh liver was taken to prepare tissue homogenate, and the supernatant was extracted after centrifugation at 3000 rpm for 10 min. HepG2 cells were collected and lysed by ultrasonic wave to prepare cell lysate. The supernatant or lysate was taken to detect the content of MDA, SOD, CAT, and GSH, and the specific operation method was carried out according to the instructions of the corresponding kit.

### 2.12 Western blot analysis

Total proteins of mouse liver and HepG2 cells were extracted with total Protein Extraction reagent, and Cytoplasmic proteins and nuclear proteins of HepG2 cells were extracted with Nuclear and Cytoplasmic Protein Extraction Kit. A total of 50 μg protein was added to 10% SDS‐PAGE, electrophoresis at low temperature for 2 h, and then transferred to PVDF membrane. After protein transfer, PVDF membranes were placed in a plastic container containing 5% skimmed milk, sealed at room temperature for 2 h. Then, dilute the first antibody and *β*‐Actin with 5% skim milk powder in a ratio of 1:1000, hybridize and incubate the PVDF membrane with the following primary antibodies: Nrf2 (16396‐1‐AP), Keap1 (10503‐1‐AP), GCLC (12601‐1‐AP), GCLM (14241‐1‐AP), HO‐1 (10701‐1‐AP), NQO1 (11451‐1‐AP), and refrigerate overnight at 4°C, respectively. The next day, dilute HRP labeled secondary antibody with 5% skim milk powder at a ratio of 1:5000, and incubate for 1 h. The protein bands were visualized by ECL reagent and analyzed by Image J software.

### 2.13 Statistical analyses

All data were presented as the mean ± SD of at least three independent experiments, and histograms were plotted using GraphPad Prism 5.0 software. Results: Statistical analysis was conducted by student's *t*‐test, and a value of *p* < .05 was considered statistically significant.

## 3 RESULTS

### 3.1 Inhibitory effects of Puerarin on APAP‐induced cell cytotoxicity

**FIGURE 1:**
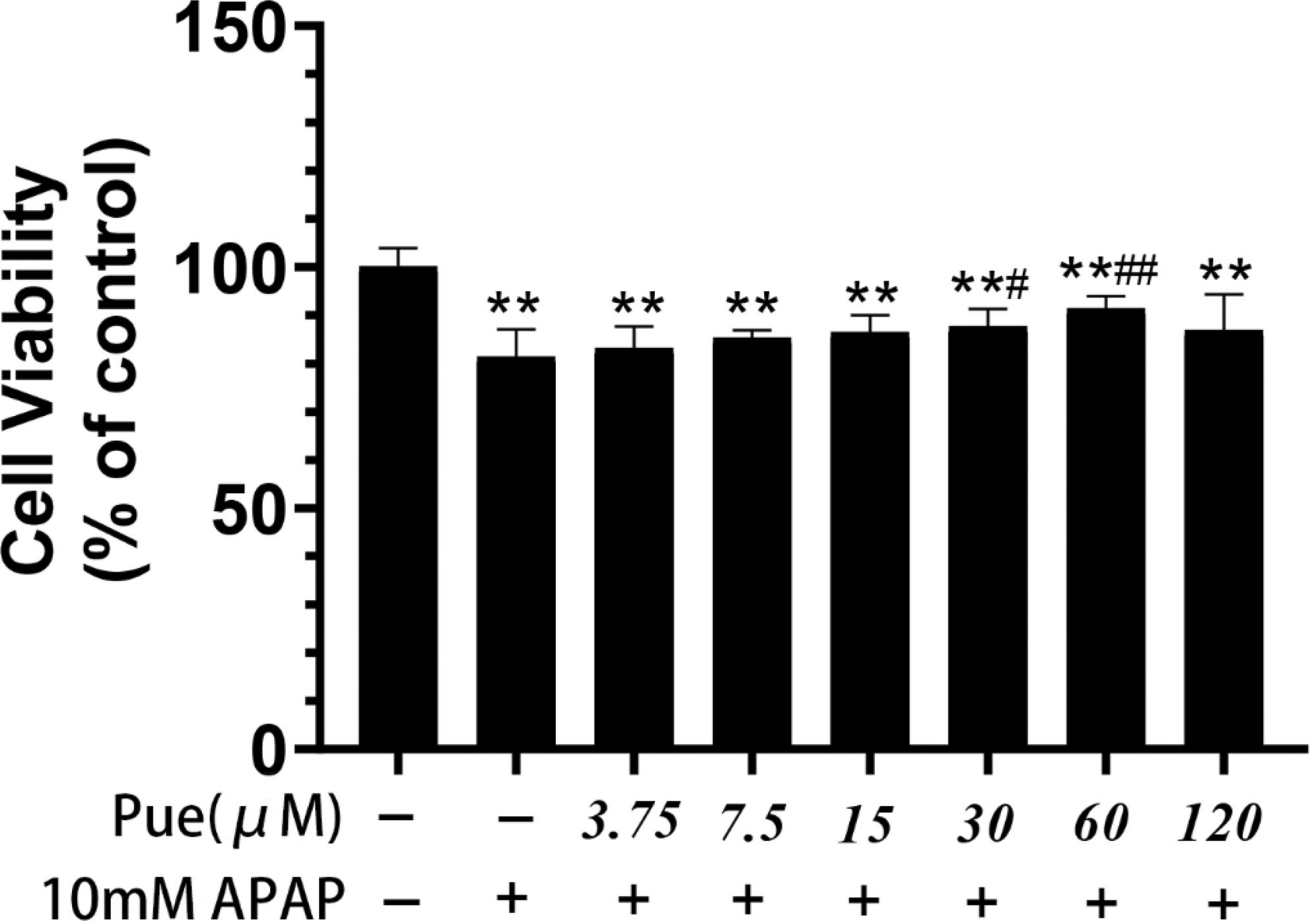
Inhibitory effects of Puerarin on APAP induced cell cytotoxicity. HepG2 cells were pretreated with different concentrations of Pue for 1 h and cultured with 10 mmol L^−1^ APAP for 24 h. Cell activity was detected by CCK‐8 assay. The data represent the mean ± SD of six independent experiments. *p < .05, **p < .01 versus Blank control group; ^#^ *p* < .05, ^##^ *p* < .01 versus APAP group.

As shown in Figure 1, Puerarin can reduce APAP‐induced HepG2 cell damage in a dose‐dependent manner in the concentration range of 60 μmol L^−1^. However, when the concentration of puerarin was less than 15 μmol L^−1^, the survival rate of HepG2 cells increased compared with APAP group but there was no statistical difference (*p* > .05). It was proved that puerarin had the hepatic‐protective potential.

### 3.2 Puerarin alleviates APAP‐induced oxidative stress in HepG2 cells

**FIGURE 2:**
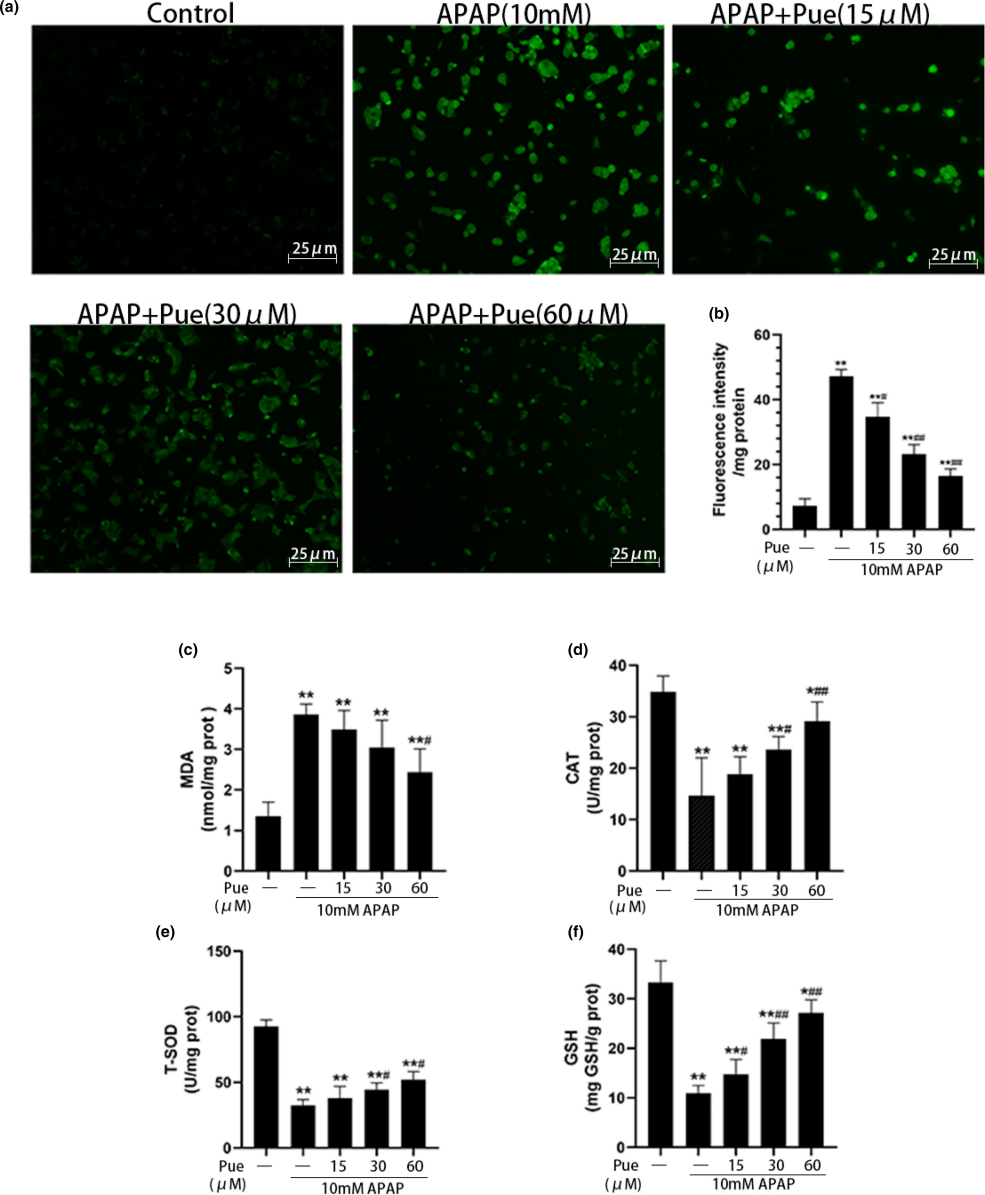
Puerarin alleviates APAP‐induced oxidative stress in HepG2 cells. HepG2 cells were pretreated with 15, 30 and 60 μmol L^−1^ Pue for 1 h, and then incubated with 10 mmol L^−1^ APAP for 24 h. DCFH‐DA fluorescent probe was added into the culture medium. Representative images were taken under a fluorescence microscope at 200× (a), and fluorescence intensity was detected with a fluorescence microplate reader (b). The cells were lysed by ultrasonic wave and the contents of MDA (c), CAT (d), T‐SOD (e) and GSH (f) in cell lysate were detected by microplate colorimetry. The data represent the mean ± SD of at least three independent experiments. *p < .05, **p < .01 versus Blank control group; ^#^ *p* < .05, ^##^ *p* < .01 versus APAP group.

Since APAP‐induced liver cell injury is mainly caused by oxidative stress, it is of great significance to study the effect of Pue on oxidative damage for evaluating its protective effect on APAP‐induced liver cell injury. The content of ROS can reflect the level of cellular oxidative stress (Ray et al., 2012). As shown in Figure 2a, by fluorescence microscope observation, compared with the control group, the ROS content in the APAP group was significantly increased. The results of present study have shown that pretreatment with different concentrations of puerarin reduced the content of reactive oxygen to different degrees. Subsequently, the fluorescence intensity was further detected by using a fluorescence microplate reader (Figure 2b). It was concluded that puerarin could inhibit ROS production in APAP‐induced damaged in HepG2 cells. Next, we examined the content of MDA, which is a marker of oxidative stress, and we observed the similar trends: Pue can alleviate APAP‐induced oxidative stress in HepG2 cells (Figure 2c). In addition, we have also detected the effect of puerarin on the antioxidant content in cells. The study showed that puerarin could significantly increase the contents of T‐SOD, CAT, and GSH in APAP‐treated HepG2 cells, thus promoting the antioxidant reaction, especially at the concentration of 60 μM, which had the best effect (Figure 2d–f). These results clearly indicate that Pue has a great antioxidant effect.

### 3.3 Effects of Puerarin on APAP induced mitochondrial dysfunction in HepG2 cells

**FIGURE 3:**
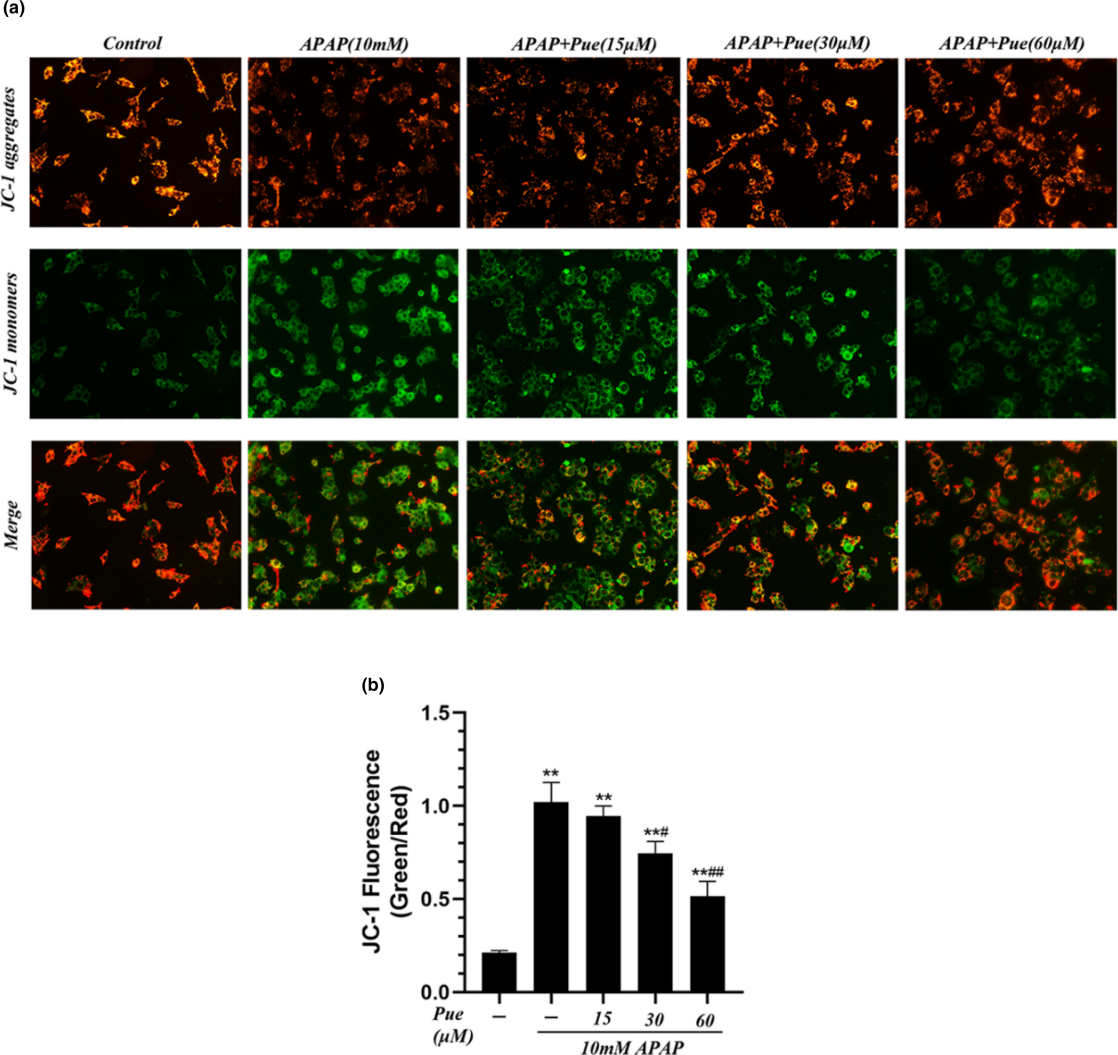
Puerarin inhibited APAP‐induced mitochondrial dysfunction in HepG2 cells. HepG2 cells were grouped with 10 mmol L^−1^ APAP and different concentrations of Pue for 24 h. (a) JC‐1 fluorescent probe was added to stain the cells, and representative images were taken under a fluorescence microscope at 200×. (b) The ratio of red‐green fluorescence was calculated. The data represent the mean ± SD of three independent experiments. *p < .05, **p < .01 versus Blank control group; ^#^ *p* < .05, ^##^ *p* < .01 versus APAP group.

It has been observed that APAP induced mitochondrial injury is the key factor for oxidative stress in the hepatocytes, and the ΔΨm reflect the extent of mitochondrial injury which is an important marker of early apoptosis (Umbaugh et al., 2021). As shown in Figure 3a, normal cells stained by JC‐1 fluorescent probe mainly emitted red fluorescence under fluorescence microscope, while HepG2 cells treated with APAP mainly presented green fluorescence. However, pretreatment of the cells with different concentrations of Pue (15, 30, 60 μmol L^−1^), has significantly reduced the APAP‐induced green fluorescence enhancement, which indicates that Pue can effectively alleviate APAP‐induced ΔΨm depolarization. Subsequently, we detected the intensity of red fluorescence and green fluorescence respectively by the luciferase plate instrument and calculated the ratio of red‐green fluorescence (Figure 3b), which further proved that puerarin can inhibit the APAP‐induced mitochondrial dysfunction in HepG2 cells.

### 3.4 Protective effect of puerarin on acute liver injury induced by APAP in mice

**FIGURE 4:**
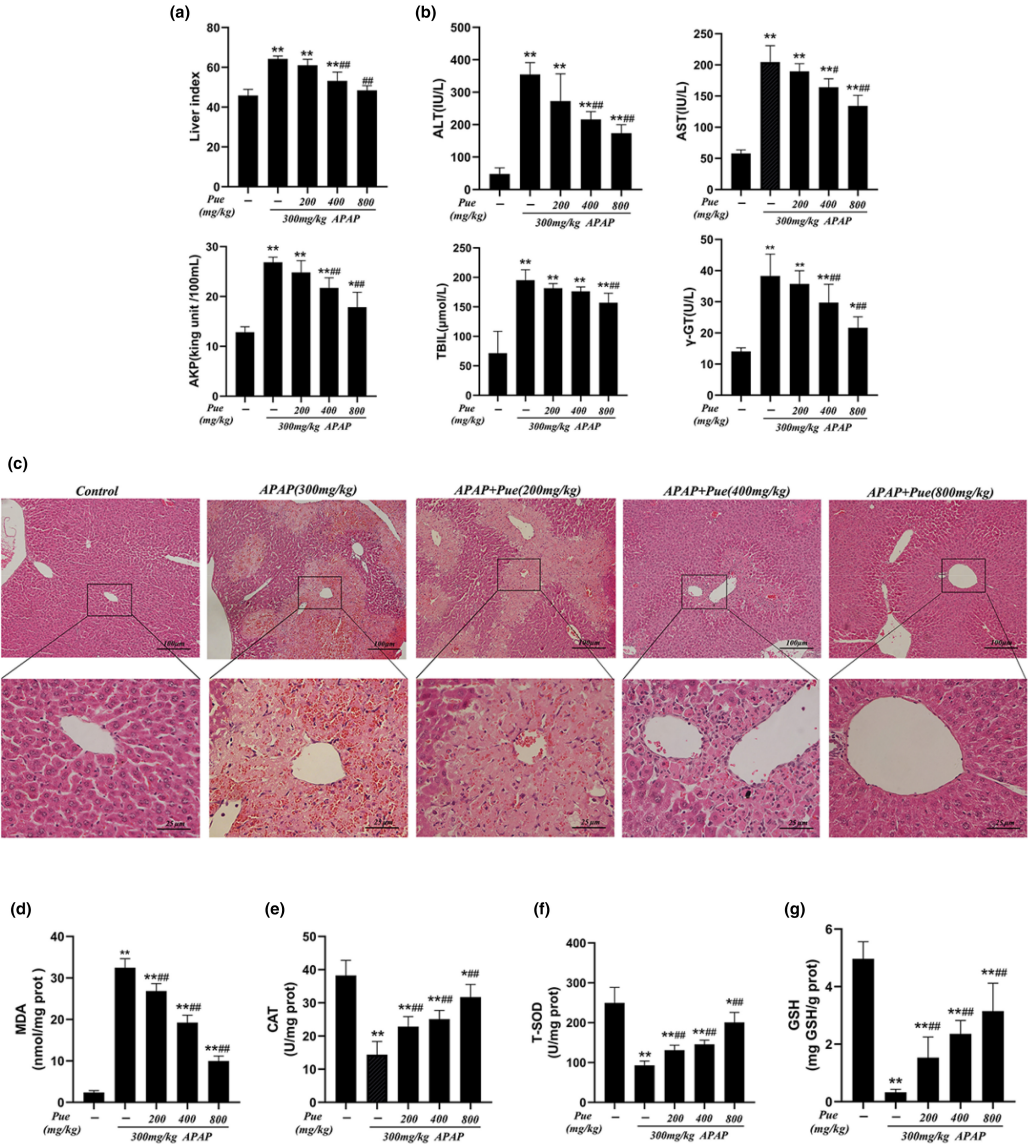
Protective effect of puerarin on acute liver injury induced by APAP in mice. After 7 days of feeding, mice were given different doses of Pue (200, 400 and 800 mg kg^−1^) for 10 days, and then, 300 mg kg^−1^ APAP was intraperitoneally injected to establish the model of acute liver injury. (a) The liver index was measured. (b) The contents of ALT, AST, TBIL and γ‐GT in serum of mice were measured. (c) The representative HE staining liver samples were observed under a 100× and 400× microscope. The liver homogenate was prepared, and the supernatant was taken after centrifugation. The contents of MDA (d), CAT (e), T‐SOD (f) and GSH (g) in supernatant were detected by microplate colorimetry. The data represent the mean ± SD, *n* = 10. *p < .05, **p < .01 versus. Blank control group; ^#^ *p* < .05, ^##^ *p* < .01 versus APAP group.

In vitro results have been demonstrated that Pue can alleviate oxidative damage induced by APAP. In order to verify whether the same effects can be obtained in vivo, we constructed a mouse model of acute liver injury using APAP. As shown in Figure 4a, compared with the blank control group, the liver index in the model group was increased significantly, and the difference was reduced to varying degrees by preventive administration of Pue (200, 400 and 800 mg kg^−1^), among which Pue_M_ and Pue_H_ showed statistically significant differences compared with the model group. In the subsequent blood biochemical tests, we found that the contents of ALT, AST, AKP, TBIL, and γ‐GT in the serum of model group mice were significantly increased, and the contents of these biochemical factors in the serum were reduced after puerarin pretreatment (Figure 4b). In order to further confirm the pathological changes in the liver tissue, HE staining was performed. As shown in Figure 4c, the liver of blank group had normal structure, and the hepatic cord around the central vein was clearly visible, however, in the model group, enhanced necrosis in the central veins surrounding cells was observed that is characterized by nuclear enrichment, karyorrhexis, karyolysis, loss of normal structure, liver blood sinus inflammatory cells infiltration. While it was observed that Puerarin pretreatment at different levels has significantly ameliorated liver injury. In addition, we also determined the antioxidant effect of puerarin in vivo. As expected, puerarin can significantly reduce the content of MDA, a marker of oxidative stress, and increase the levels of CAT, T‐SOD and GSH in liver (Figure 4d–g). These results suggest that puerarin has a protective effect on acute liver injury induced by APAP.

### 3.5 Puerarin activates the Nrf2 signaling pathway by inhibiting Keap1

**FIGURE 5:**
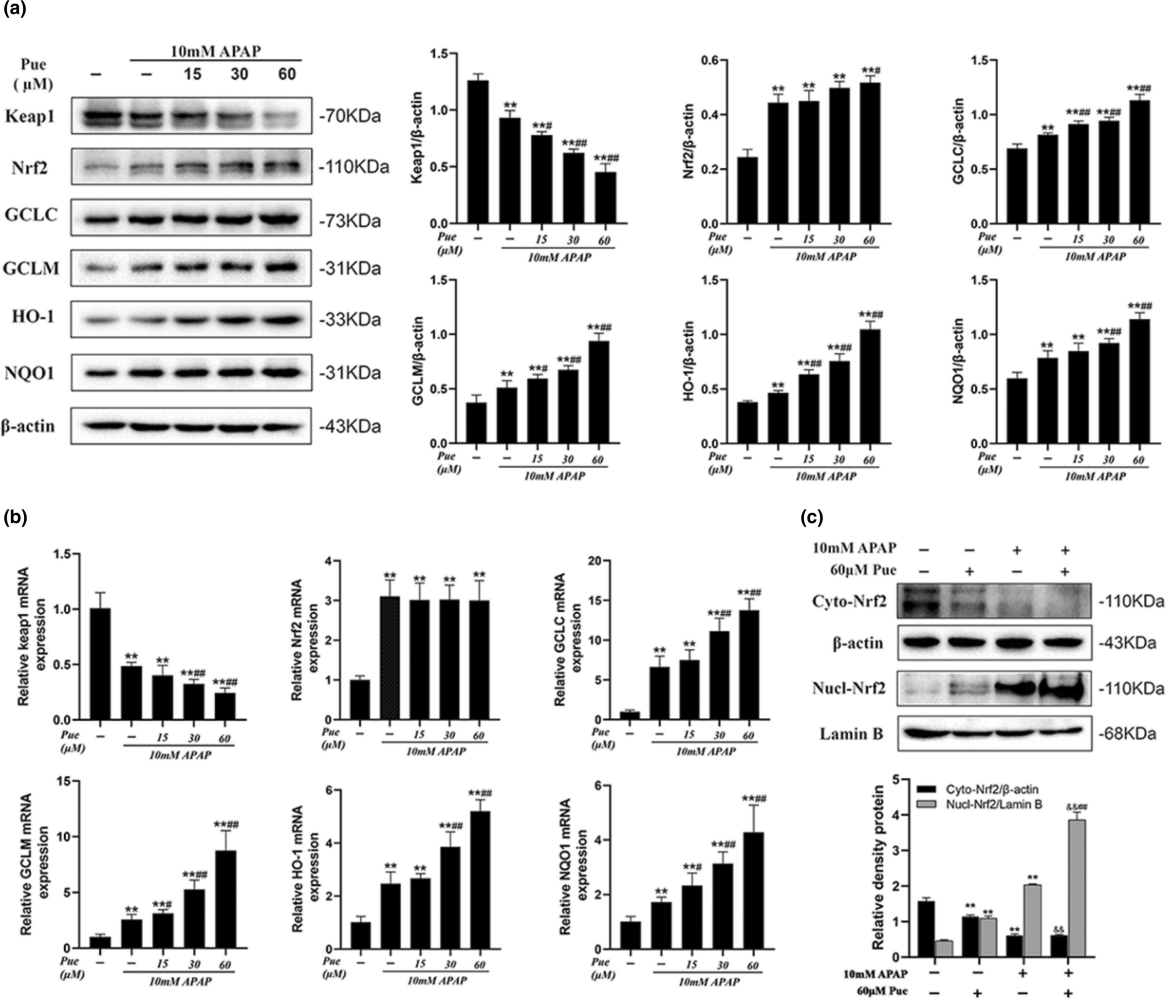
Puerarin activates the Nrf2 signaling pathway by inhibiting Keap1. HepG2 cells were grouped with 10 mmol L^−1^ APAP and different concentrations of Pue for 24 h; Mice were given different doses of Pue (200, 400 and 800 mg kg^−1^) for 10 days, and then, 300 mg kg^−1^ APAP was intraperitoneally injected to establish the model of acute liver injury. (a) The protein contents of Keap1, Nrf2, GCLC, GCLM, HO‐1 and NQO1 in HepG2 cells lysates were analyzed by Western blot analysis. (b) Total RNA was extracted from HepG2 cells, and quantitative real‐time PCR was used to detect the mRNA expression levels of KEAP1, Nrf2, GCLC, GCLM, HO‐1, and NQO1. (c) The protein expression levels of Nrf2 in cytoplasm and nucleus were analyzed by Western blot. Expression levels of Keap1, Nrf2, GCLC, GCLM, HO‐1 and NQO1 in mouse liver were analyzed by Western blot. The data represent the mean ± SD of at least three independent experiments. *p < .05, **p < .01 versus Blank control group; ^#^ *p* < .05, ^##^ *p* < .01 versus APAP group; ^&^ *p* < .05, ^&&^ *p* < .01 versus Pue‐treated group.

Nrf2 signaling pathway is the regulatory center of the oxidative stress response in organisms and can regulate the expression of a variety of antioxidant reactive‐related genes or enzymes. Therefore, we were interested in finding out the effect of puerarin on the Nrf2 signaling pathway of APAP‐induced oxidative damage to HepG2. As shown in Figure 5a, compared with APAP group, Pue treatment reduced the expression of Keap1 in oxygen‐damaged HepG2 cells and increase the expression of GCLC, GCLM, HO‐1, and NQO1 downstream of Nrf2 signaling pathway, while only 60 μM of Pue could significantly enhance the expression of Nrf2. Interestingly, we found that Pue did not promote the transcription of Nrf2, while the transcription levels of Keap1, GCLC, GCLM, HO‐1, and NQO1 showed the same trend as the protein levels (Figure 5b). On this basis, we detected the expression of Nrf2 in cytoplasm and nucleus of HepG2 cells, respectively. The results showed that the treatment of Pue alone could significantly promote the nuclear migration of Nrf2, and this phenomenon was more obvious in APAP‐induced oxidative damage cells (Figure 5c).

**FIGURE 6:**
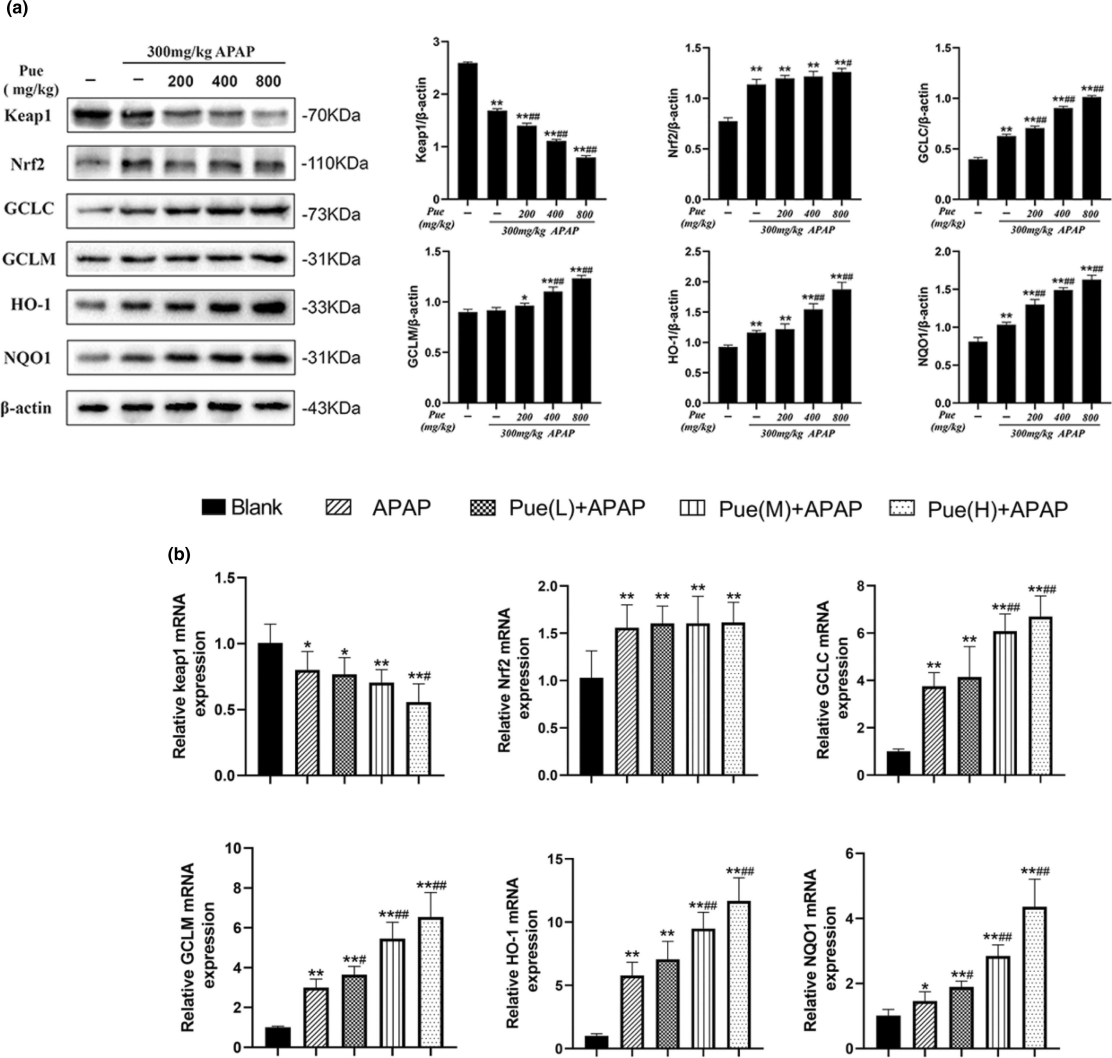
Puerarin activates the Nrf2 signaling pathway by inhibiting Keap1. Mice were given different doses of Pue (200, 400 and 800 mg kg^−1^) for 10 days, and then 300 mg kg^−1^ APAP was intraperitoneally injected to establish the model of acute liver injury. (a) The protein expression levels of Keap1, Nrf2, GCLC, GCLM, HO‐1 and NQO1 in mouse liver were analyzed by Western blot. (b) Total RNA was extracted from mouse liver, and Quantitative real‐time PCR was used to detect the mRNA expression levels of KEAP1, Nrf2, GCLC, GCLM, HO‐1 and NQO1. The data represent the mean ± SD of at least three independent experiments. *p < .05, **p < .01 versus Blank control group; ^#^ *p* < .05, ^##^ *p* < .01 versus APAP group; ^&^ *p* < .05, ^&&^ *p* < .01 versus Pue‐treated group.

In order to verify whether puerarin has the same effect in vivo, we investigated the effect of puerarin on Nrf2 signaling pathway in APAP‐induced acute liver injury mice. As shown by western blot analysis (Figure 6a), compared with the blank group, the expression of Keap1 protein was significantly reduced in mice with acute liver injury; meanwhile, the expression of Nrf2, GCLC, HO‐1, and NQO1 increased significantly, while GCLM showed an upward trend but no statistical difference. Compared with the APAP group, the expression of Keap1 was decreased and the expression of other proteins were further increased after pretreatment with Pue in advance. It is worth mentioning that the expression of Nrf2 was significantly increased only in Pue_H_. On this basis, we detected the expression of Nrf2 in cytoplasm and nucleus of mice liver, respectively. The results showed that the treatment of Pue alone could significantly promote the nuclear migration of Nrf2, and this phenomenon was more obvious in APAP‐induced oxidative damage liver cells (Figure 6b). On the basis of vivo results, we have consistently concluded that Pue activates the Nrf2 signaling pathway mainly by inhibiting Keap1.

Taken together, these findings we concluded that Pue can promote the nuclear translocation of Nrf2 by inhibiting the expression of Keap1, thereby activating the expression of GCLC, GCLM, HO‐1, and NQO1, exerting antioxidant effects and alleviating the hepatocyte injury caused by APAP.

## 4 DISCUSSION

The liver is one of the most important organs of the organism, serving as the two most important functions of metabolism and detoxification. Drug‐induced liver injury is a common type of liver disease and has become a serious public health problem worldwide (Ramachandran & Jaeschke, 2019). Based on the antioxidant function of puerarin, in this study, we have systematically evaluated the protective effect of puerarin on hepatotoxicity induced by APAP in vivo and in vitro.

Organ index is one of the biological characteristics that used to measure and reflects the functional state of animals, which can be used as evidence to evaluate histopathological changes, and it is also a sensitive index to judge the toxicity of drugs (Bailey et al., 2004). Due to the low bioavailability of puerarin in oral administration (El Desoky et al., 2022; Zhang, 2019), mice were treated with 200, 400, 800 mg kg^−1^ Pue for 10 days in advance and then injected intraperitoneally with 300 mg kg^−1^ APAP to establish the model of acute liver injury. Interestingly, we found that Pue can significantly reduce liver index in APAP injected mice. In order to further confirm the pathological changes of liver tissue, the liver sections were observed by HE staining in this experiment. The results showed that Pue could reduce the necrosis of liver cells around the central vein to a certain extent.

For liver diseases, blood biochemical test can reflect the changes of liver function to a large extent, so it also has a particularly important application value. Serum levels of ALT, AST, AKP, γ‐GT, and TBIL are the most common indicators of liver function evaluation (Kwo et al., 2017; Woreta & Alqahtani, 2014). We found that compared with the blank control group, the five liver function related indexes in the APAP group were significantly changed, and ALT and AST index increased the most, which was consistent with the blood biochemical characteristics of acute liver injury caused by acetaminophen (Andrade et al., 2019). The serum contents of ALT, AST, AKP, γ‐GT, and TBIL in mice pretreated with different doses of Pue were reduced in a dose‐dependent manner. These results proved that puerarin has a protective effect on APAP‐induced acute liver injury.

Oxidative stress is the key cause of liver injury caused by APAP. GSH is a kind of low molecular scavenger, which can be used to measure the antioxidant capacity of the organism (Townsend et al., 2003). Since the toxic mechanism of oxidative stress induced by APAP is the depletion of GSH, which makes it particularly important to detect the GSH content in the liver. SOD and CAT are important components of the biological antioxidant enzyme system, which can remove oxygen free radicals produced by metabolism in cells and protect cells from the toxicity of peroxides (Ighodaro & Akinloye, 2018). MDA is the product of lipid peroxidation and can indirectly reflect the degree of cell damage (Całyniuk et al., 2016). Therefore, GSH, SOD, CAT, and MDA can be used as indexes to evaluate liver oxidative damage. Both in vivo and in vitro experimental results showed that Pue treatment increased the contents of GSH, SOD and CAT in liver and HepG2 cells, and reduce the content of MAD. Subsequently, the experiment for determination of ROS in HepG2 cells was performed. It was observed that Pue treatment has significantly reduced the APAP induced ROS increase, which proved that Pue could effectively alleviate the oxidative stress. Oxidative stress is linked to mitochondrial dysfunction (Ott et al., 2007). The decrease of ΔΨm indicates mitochondrial dysfunction and is also one of the early markers of apoptosis. We used JC‐1 fluorescent probe to detect ΔΨm, and as expected, puerarin could alleviate the decrease of ΔΨm caused by APAP, that is, alleviate mitochondrial damage.

Nrf2 as a key coordinating factor of anti‐oxidative stress, plays an indispensable role in improving and alleviating acute liver injury. Crucially, among Nrf2‐regulated proteins, GCLC and GCLM act as the catalytic and regulatory subunits of γ‐GCS, respectively, and their expression determines the activity of γ‐GCS, which are rate‐limiting enzymes for GSH synthesis (Lu, 2009). Therefore, Nrf2 signaling pathway may become a potential target for the treatment of APAP‐induced acute liver injury. Keap1 is the upstream negative regulator of Nrf2, and has an inhibitory effect on the nuclear translocation of Nrf2 (Suzuki et al., 2013). In this study, we investigated the effect of Pue on the Nrf2 signaling pathway. Our experimental conclusion showed that Pue can inhibit the expression of Keap1 to promote the nuclear migration of Nrf2, and activate the expressions of GCLC, GCLM, HO‐1, and NQO1, so as to promote the production of cell antioxidants and alleviate mitochondrial damage. Subsequent in vivo studies supported this view. Interestingly, we found that APAP can also activate the Nrf2 signaling pathway, which is consistent with existing report (Shen et al., 2018). This is because cells under the state of oxidative stress will spontaneously activate the Nrf2 signaling pathway to play the role of anti‐oxidative damage, while Pue can further promote the antioxidant reaction without causing cell damage.

By reason of the foregoing, these data suggest that Pue has excellent antioxidant activity both in vivo and in vitro, may alleviates APAP‐induced liver injury through activation of Nrf2 signaling pathway. Although the clinical practicality of puerarin is limited by its low bioavailability, these problems will be solved with the development of microemulsion and nanoparticle technology (Luo et al., 2011; Wu et al., 2018). We have reason to believe that Pue has the possibility of becoming a candidate drug for preventing liver injury and has a broad application prospect.

## 5 CONCLUSION

To sum up, Pue can activate the antioxidant reaction by inhibiting the expression of Keap1, which can control the nuclear migration of Nrf2, thereby enhancing the activities of GSH and various antioxidant enzymes to reduce the accumulation of ROS and alleviate mitochondrial damage, thus playing a protective role against the liver injury caused by APAP. Therefore, Pue may be considered as a potential complementary and alternative therapy for the prevention of APAP‐induced acute liver injury.

## AUTHOR CONTRIBUTIONS

Wanhai Zhou: Conceptualization, Formal analysis, Investigation, Methodology, Resources, Date curtin, Validation, Visualization, Writing‐original draft. Heng He: Conceptualization, Formal analysis, Investigation, Methodology, Resources, Date curtin, Validation, Visualization, Writing‐original draft, Writing‐review & editing. Qin Wei & Litao Che: Visualization, Formal analysis, Investigation, Methodology. Xin Zhao: Data curation, Formal analysis, Validation, Visualization. Wenwen Liu & Yue Yan: Validation, Visualization. Liangqing Hu & Yonghua Du: Visualization, Formal analysis, Investigation, Supervision. Yongkang Shuai & Li Yang: Validation, Visualization, Writing‐review & editing. Zhongqiong Yin & Ruizhang Feng: Conceptualization, Formal analysis, Funding acquisition, Investigation, Methodology, Project administration, Resources, Supervision, Writing‐review & editing.

## CONFLICT OF INTEREST STATEMENT

The authors declare that they have no known competing financial interests or personal relationships that could have appeared to influence the work reported in this paper.

## ETHICS STATEMENT

All procedures involving animals and their care in this study were approved (No. 20190604) by the Ethics Committee of Sichuan Agricultural University according to the Regulation of Experimental Animal Management (State Scientific and Technological Commission of the People's Republic of China, No. 2, 1988) and The Interim Measures of Sichuan Province Experimental Animal Management (Science and Technology Bureau of Sichuan, China, No. 25, 2013).

## Data Availability

All datasets presented in this study are include in the article/supplementary material.
